# Novel chemical synthesis and characterization of copper pyrovanadate nanoparticles and its influence on the flame retardancy of polymeric nanocomposites

**DOI:** 10.1038/srep25231

**Published:** 2016-05-04

**Authors:** Maryam Ghiyasiyan-Arani, Maryam Masjedi-Arani, Davood Ghanbari, Samira Bagheri, Masoud Salavati-Niasari

**Affiliations:** 1 Institute of Nano Science and Nano Technology, University of Kashan, Kashan, P. O. Box.87317-51167, I. R. Iran; 2 Young Researchers and Elite Club, Arak Branch, Islamic Azad University, Arak, Iran; 3 Nanotechnology & Catalysis Research Centre (NANOCAT), IPS Building, University of Malaya, 50603 Kuala Lumpur, Malaysia

## Abstract

In this work, copper pyrovanadate (Cu_3_V_2_O_7_(OH)_2_(H_2_O)_2_) nanoparticles have been synthesized by a simple and rapid chemical precipitation method. Different copper-organic complexes were used to control the size and morphology of products. The morphology and structure of the as-synthesized products were characterized by X-ray diffraction (XRD), scanning electron microscopy (SEM), transmission electron microscopy (TEM), Fourier transform infrared (FT-IR) spectrum, electron dispersive X-ray spectroscopy (EDX), thermal gravimetric analysis (TGA), differential thermal analysis (DTA) and photoluminescence (PL) spectroscopy. The influence of copper pyrovanadate nanostructures on the flame retardancy of the polystyrene, poly vinyl alcohol and cellulose acetate was studied. Dispersed nanoparticles play the role of a magnetic barrier layer, which slows down product volatilization and prevents the flame and oxygen from the sample during decomposition of the polymer. Cu_3_V_2_O_7_(OH)_2_(H_2_O)_2_ is converted to Cu_3_V_2_O_8_ with an endothermic reaction which simultaneously releases water and decrease the temperature of the flame region.

Considerable attention has been paid to the synthesis of the nanostructured materials during the last decade because of their extensive properties and wide range of applications^1^,^2^. Nanostructured copper vanadates are widely used in applications such as lithium ion batteries^3^,^4^,^5^,^6^,^7^, antibacterial additive^8^, Ion-exchange materials^9^, electrochemical properties^10^,^11^,^12^ and catalyst^13^,^14^. There are different types of mixed oxides based on vanadium and copper such as (CuV_2_O_6_)^15^,^16^, (Cu_2_V_2_O_7_)^17^,^18^, (Cu_3_V_2_O_8_)^19^,^20^, (Cu_5_V_2_O_10_)^21^ and Cu_3_(OH)_2_V_2_O_7_·nH_2_O^7^,^22^. Volborthite, Cu_3_V_2_O_7_(OH)_2_(H_2_O)_2_, is an interesting layered crystalline material that consists of a copper layer, in octahedral coordination with oxygen, joined by vanadium tetrahedral in a coordinated layer. Various methods for the synthesis of copper vanadate have been studied such as laser ablation^23^, hydrothermal^12^,^22^, sol-gel^24^ and co-precipitation^10^. Precipitation method was used for preparation of Cu_3_V_2_O_7_(OH)_2_(H_2_O)_2_ (CVO) nanostructures. The precipitation method is a simple, fast and cost effective synthetic procedure for preparing of copper pyrovanadate nanostructures. To optimize the size, morphology and also properties of Cu_3_V_2_O_7_(OH)_2_(H_2_O)_2_ nanostructures, copper-organic complexes ([Cu(en)_2_]SO_4_, [Cu(pn)_2_]SO_4_, [Cu(TETA)]SO_4_ and [Cu(TEPA)]SO_4_) were chosen as Cu precursor. It has been demonstrated that these kinds of metal-organic complexes have an effective role on size controlling, morphology of the final products^25^. Herein, we report a simple and surfactant-free route for the synthesis of copper pyrovanadate nanostructures via the chemical precipitation method from 3:2 molar ratio of copper-organic complexes and NH_4_VO_3_. Polymeric nanocomposites have recently gained much attention because adding a small amount of nanostructure to a polymeric matrix can lead to improvement properties of the matrix^26^,^27^,^28^,^29^. The principal profits of these compounds over many metallic alloys are corrosion resistance, low density and thermal insulation. However the main disadvantage of polymeric compounds is high flammability. Using of the most traditional and toxic flame retardants like halogenated and aromatic compounds are forbidden with respect to the environmental considerations. Herein, the influence of copper pyrovanadate nanostructures on the flame retardancy of the polymeric matrix nanocomposites was studied.

## Results and Discussion

**Figure 1:**
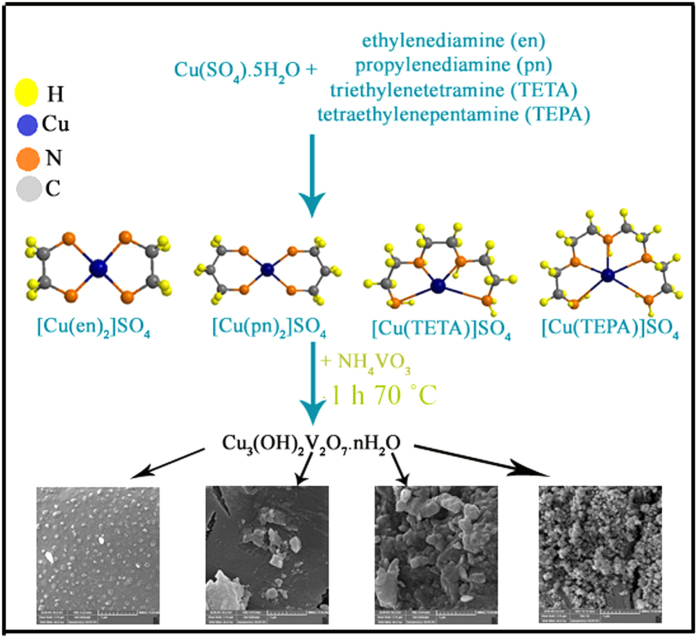
Schematic depiction for the preparation of Cu_3_V_2_O_7_(OH)_2_(H_2_O)_2_ nanoparticles.

**Figure 2:**
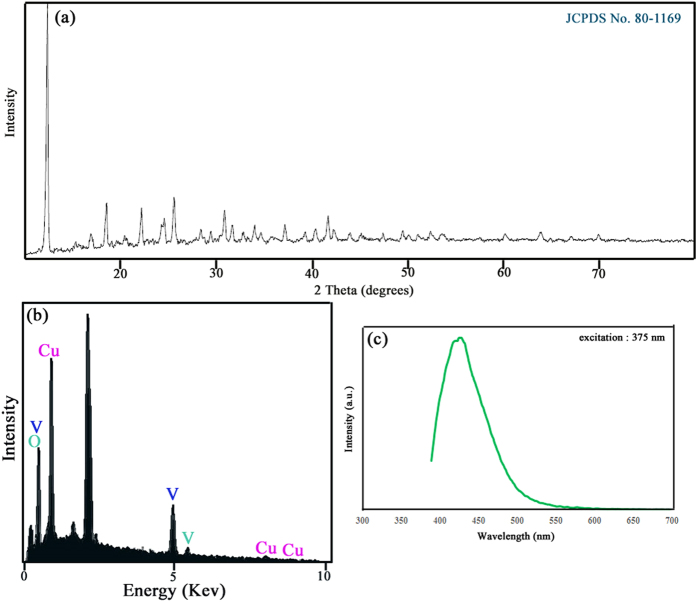
(**a**) XRD, (**b**) EDS patterns, (**c**) Photoluminescence spectrum of pure Cu_3_V_2_O_7_(OH)_2_(H_2_O)_2_ nanoparticles (sample No.4) and (**d**) Magnetization versus applied magnetic field at room temperature for the Cu_3_V_2_O_7_(OH)_2_(H_2_O)_2_ nanoparticles (sampleNo. 4).

Figure 1 show schematic diagram of formation of Cu_3_V_2_O_7_(OH)_2_(H_2_O)_2_ nanoparticles. The preparation conditions for the synthesis of Cu_3_V_2_O_7_(OH)_2_(H_2_O)_2_ nanoparticles have been illustrated in Table 1. Figure 2a shows XRD pattern of copper pyrovanadate sample prepared with copper-organic complex of [Cu(en)_2_]SO_4_ (sample No.1). All diffraction peaks were indexed to pure Monoclinic phase of Cu_3_(OH)_2_V_2_O_7_.nH_2_O with space group of C2/m and cell constants a = 10.6060 Å, b = 5.8740 Å, and c = 7.2130 Å (JCPDS Card No.80–1169). The crystallite diameter (D_c_) of CVO nanostructures has been found to be 45 nm. EDS analysis measurement was employed to investigate the chemical composition and purity of the copper pyrovanadate nanoparticles. EDS analysis of nanoparticles (sample No. 4) is illustrated in Fig. 2b and confirms the presence of Cu, V and O in the sample. According to EDS results, atomic percentage of elements are 4.03% Copper, 15.06% Vanadium and 80.91% Oxygen. Photoluminescence (PL) spectrum of copper pyrovanadate nanoparticles were obtained at room temperature with an excitation wavelength of 375 nm, and is shown in Fig. 2c. The PL spectrum consists of one strong peak at 425 nm. Band gap of as-synthesized sample was obtained to 2.91 eV, which shows blue shift compared with Cu_3_V_2_O_7_(OH)_2_(H_2_O)_2_ nanowires (1.94–2.22 eV)^30^.

Hysteresis loop and magnetic property of Cu_3_V_2_O_7_(OH)_2_(H_2_O)_2_ nanostructures (sample No.4) is shown in Fig. 2d. The saturation magnetization (M_S_) and coercivity (Hc) of Cu_3_V_2_O_7_(OH)_2_(H_2_O)_2_ nanostructures (sample No.4) are about 0.0117 emug^−1^ and 137 Oe respectively. The behavior of copper pyrovanadate nanoparticles was changed from paramagnetic to ferromagnetic at fields of lower than 1500 Oe. The as-prepared sample shows a twofold behavior, ferromagnetic behavior in low fields and paramagnetic behavior in high fields.

**Figure 3:**
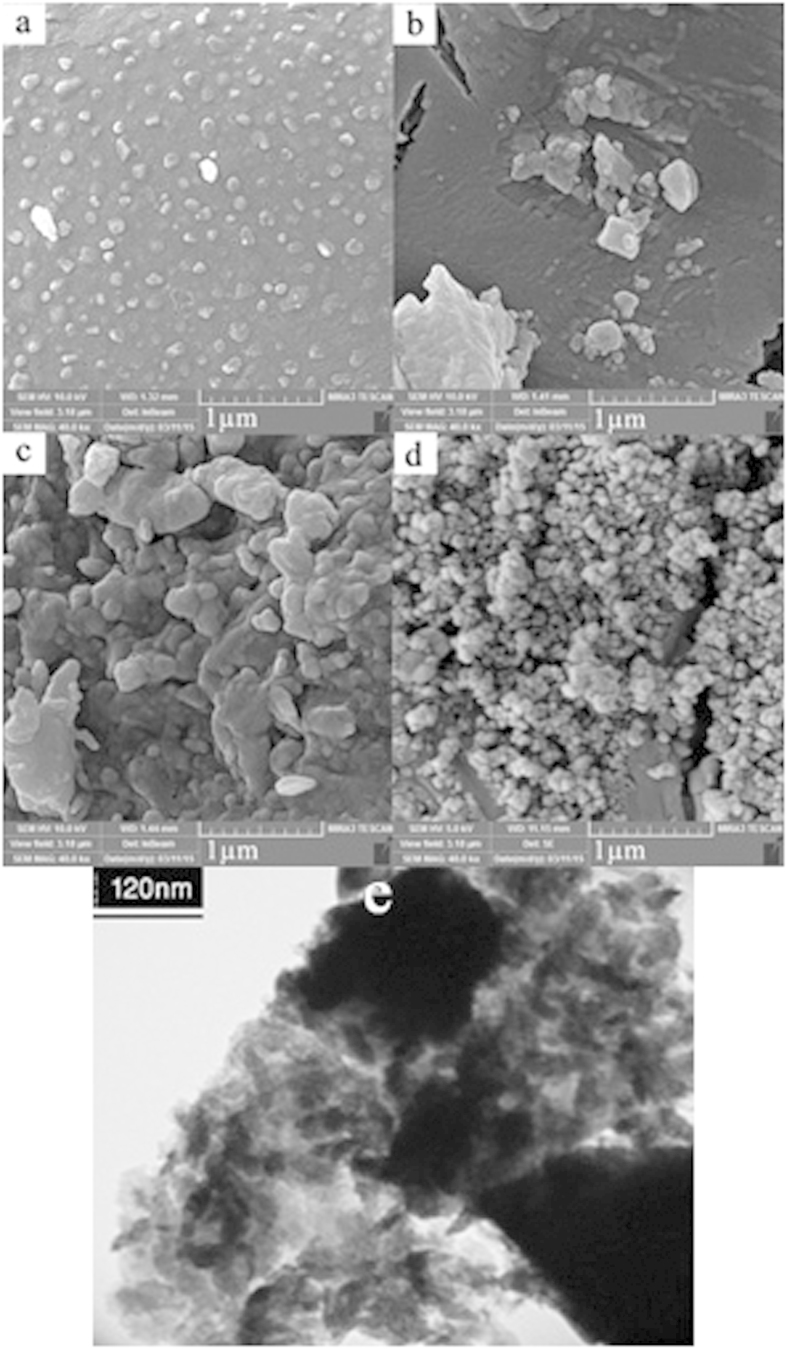
SEM images of the Cu_3_(OH)_2_V_2_O_7_.nH_2_O nanoparticles with different copper-organic complexes (**a**) [Cu(en)_2_]SO_4_, (**b**) [Cu(pn)_2_]SO_4_, (**c**) [Cu(TETA)]SO_4_, (**d**) [Cu(TEPA)]SO_4_, (**e**) CuSO_4_.5H_2_O, f) TEM image of nanostructures obtained with [Cu(TEPA)]SO_4_ and g) Particle size distribution of samples Nos. 1 and 4.

The influence of different copper-organic complexes on the morphology and particle size of copper pyrovanadate samples were investigated by FESEM. Fig. 3a–d show SEM images of copper pyrovanadate samples prepared in presence of [Cu(en)_2_]SO_4_, [Cu(pn)_2_]SO_4_, [Cu(TETA)]SO_4_ and [Cu(TEPA)]SO_4_ (sample Nos. 1–4) respectively. These results show that using [Cu(pn)_2_]SO_4_ and [Cu(TETA)]SO_4_ complexes lead to synthesis of agglomerated products. However, by using [Cu(en)_2_]SO_4_ and [Cu(TEPA)]SO_4_ complexes, nanostructure products are obtained. The particle size of copper pyrovanadate nanostructures obtained with [Cu(TEPA)]SO_4_ complex are smaller than those produced by [Cu(en)_2_]SO_4_ complex. It is observed that with increasing steric hindrance of complex, a decrease in particle size occurs.

In Fig. 3e, CuSO_4_.5H_2_O was used as copper source and other reaction parameters remained unchanged. The products obtained from CuSO_4_.5H_2_O salt as a blank test lead to larger particles than those obtained from Cu-organic complexes. The bulk and agglomerated Cu_3_V_2_O_7_(OH)_2_(H_2_O)_2_ products were synthesized by surfactant-free reactions^7^,^30^,^31^.

The amine ligands in Cu-organic complexes, can act as a capping agent and provide steric hindrance. These results show that using [Cu(TEPA)]SO_4_ complex lead to synthesis of nano-products. It was observed that with increasing steric hindrance of complex, a decrease in particle size was appeared^32^. Results of particle size of products are shown at Table 1. The precise morphology and particle size of prepared copper pyrovanadate nanostructures with [Cu(TEPA)]SO_4_ complex was elucidated by TEM. Figure 3f shows the TEM image of sample No. 4 which contains nanoparticles with size of less than 100 nm.

Typical histograms of the particle diameters for the samples obtained using [Cu(en)_2_]SO_4_ and [Cu(TEPA)]SO_4_ are compared in Fig. 3g, respectively. By comparing the particle size distribution of the products, Cu_3_V_2_O_7_(OH)_2_(H_2_O)_2_ that was prepared using [Cu(TEPA)]SO_4_ have smaller particle size distribution (30–70 nm). By using Cu-organic complexes due to the presence of amine ions in reaction medium, pH of reaction was upper than 6. Whereas, Zhang *et al.* used NH_3_ as basic agent in synthesis of Cu_3_V_2_O_7_(OH)_2_(H_2_O)_2_ by using CuSO_4_.5H_2_O and NH_4_VO_3_ precursors^10^. Moreover, Cu-organic complexes lead to formation of nanostructures and there is no need to use of surfactants. Sun *et al.* used CTAB as a surfactant for preparing of flower-like Cu_3_V_2_O_7_(OH)_2_.2H_2_O microstructures^31^.

**Figure 4:**
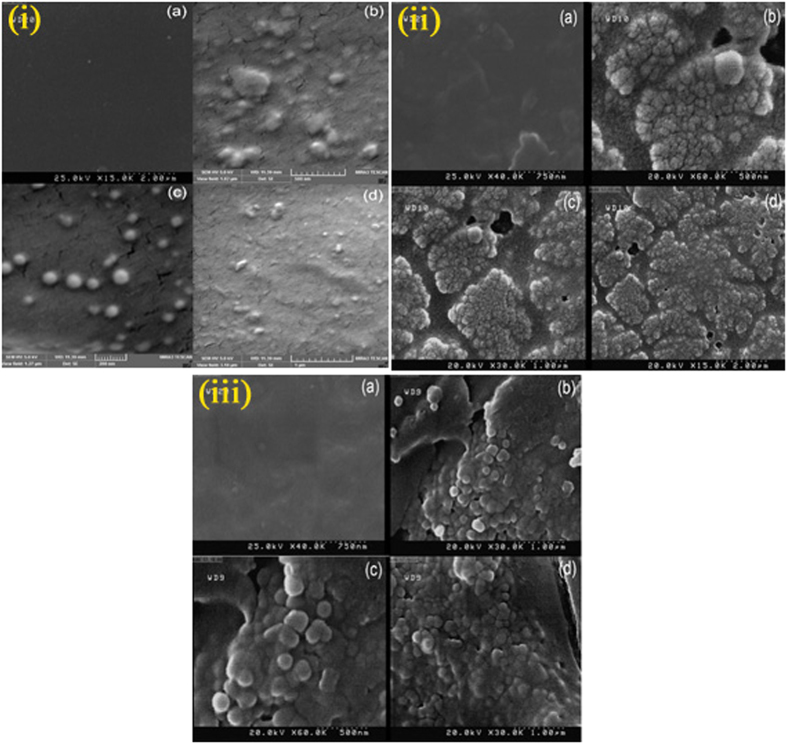
SEM images of the (i): (**a**) pure PVA (b,c,d) PVA nanocomposite, ii: (**a**) pure PS (**b–d**) PS nanocomposite and (iii): (**a**) pure CA (**b–d**) CA nanocomposite.

**Figure 5:**
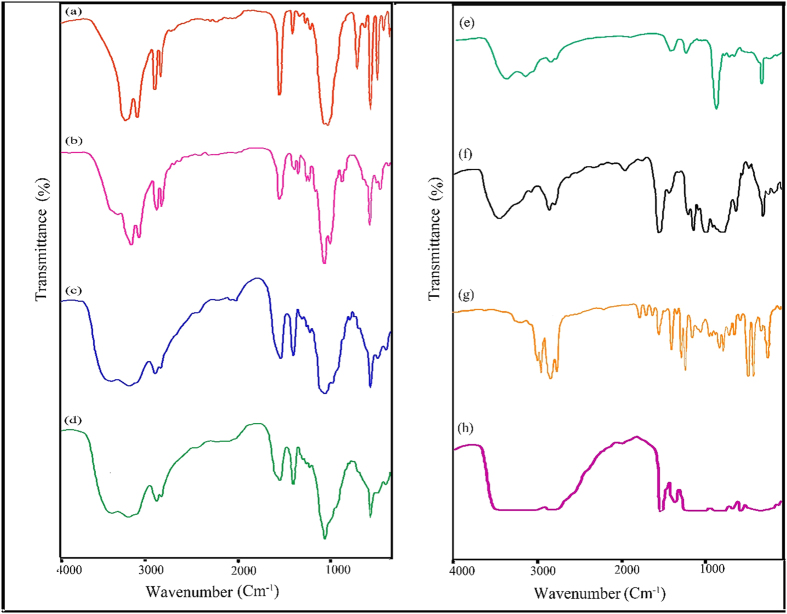
FT-IR spectra of (**a**) [Cu(en)_2_]SO_4_, (**b**) [Cu(pn)_2_]SO_4_, (**c**) [Cu(TETA)]SO_4_, (**d**) [Cu(TEPA)]SO_4_, (**e**) CVO nanoparticles, (**f**) CA, (**g**) PS and (**h**) PVA.

Figure 4i(a) shows SEM image of pure poly vinyl alcohol; pure polymers show smooth and flat surfaces and only some cracks due to interaction of polymer and electron beam were observed and also Fig. 4i(b–d) illustrate SEM images PVA-CVO nanocomposite in three different magnifications that obviously confirm presence of spherical nanoparticles in the polymeric nanocomposites. Figure 4ii(a) shows SEM image of flat poly styrene surface and Fig. 4ii(b–d) illustrate SEM images PS-CVO in approve the presence of nanoparticles in the polymeric nanocomposites. Because of hydrophilic property of CVO and hydrophobic of poly styrene there is an expected agglomeration in PS-CVO nanocomposite. Figure 4iii(a) also exhibit SEM image of smooth cellulose acetate Fig. 4iii(b–d) give SEM images of CA-CVO that appropriately show nanoparticles in the polymeric nanocomposites. Figures 5(a–d) show FT-IR spectra of the [Cu(en)_2_]SO_4_, [Cu(pn)_2_]SO_4_, [Cu(TETA)]SO_4_ and [Cu(TEPA)]SO_4_ complexes, respectively. In these spectra, the absorption around 3230 cm^−1^ can be assigned to the stretching vibration of the N-H of amine groups. Two absorption bands around 2940 and 2880 cm^−1^ can be assigned to the symmetry stretching and asymmetry stretching mode of the CH_2_ groups, respectively. The bands around 3300, 1610, 930 and 620 cm^−1^ can be related to water molecules. The absorption bands at 1440 and 1054 cm^−1^ can be attributed to the C–H bending vibration and C–N stretching vibration, respectively. The bands at 504 and below 480 cm^−1^ is assigned to m(Cu–O) and m(Cu–N) vibration, respectively. FT-IR spectrum of CVO is shown in Fig. 5(e–h) absorptions at 450 and 3249 cm^−1^ are related to Cu-O and O-H bonds respectively. Figure 5f illustrates spectrum of CA–CVO nanocomposite, absorptions at 1083 and 3470 cm^−1^ are attributed to C-O and hydroxyl bonds respectively and peaks at 1440 and 1600 cm^−1^ are responsible to C = O bonds. Spectrum of PS–CVO nanocomposite is depicted in Fig. 5g in which the peak absorptions at 2922 and 3028 cm^−1^ are related to aliphatic and aromatic C–H bonds, respectively. Spectrum of PVA–CVO nanocomposite is depicted in Fig. 5h which the peak at 1090 cm^−1^ is related to V-O bond. Peaks at 1561 and 1747 cm^−1^ are correspond to C = O bonds and also absorptions at 1083 and 3470 cm^−1^ are attributed to C-O^33^,^34^,^35^.

**Figure 6:**
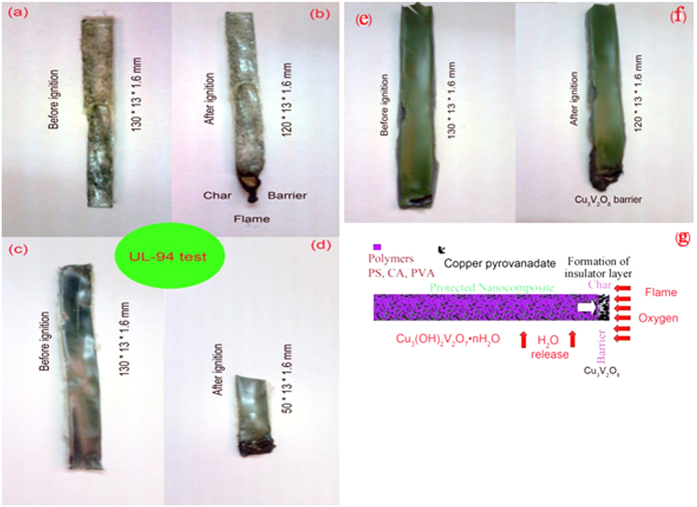
UL 94 analysis of the (**a,b**) CA (**c,d**) PS (**e,f**) PVA (**g**) schematic of barrier formation.

The effect of inorganic nanostructure on the flame retardant properties of the polymers has been considered using UL-94 test. The outcomes show that CVO additives can enhance the flame retardant property of the polymeric matrices. *Ex-situ* products were easily obtained and made from two separated phases of copper pyrovanadate and polymeric matrices. The main challenge in the synthesis of nanocomposite is dispersion of inorganic phase in organic matrix. Hydroxyl group in copper pyrovanadate nanoparticles lead to suitable interaction with hydrophilic polymers like vinyl alcohol and cellulose acetate matrices. The results of UL-94 tests for poly styrene and cellulose acetate nanocomposites are NC and V-1 respectively (Fig. 6a–d). The worst result was obtained for PS-CVO because of incompatibility of hydrophobic polystyrene and hydrophilic CVO. The result of UL-94 tests for poly vinyl alcohol nanocomposites is V-0. HO…Cu-V-O-Cu…OH compound and other precursors as binder are compatible with polymeric matrices. The enhancement of flame retardancy of nanocomposite is due to formation of effective barrier layer of Cu-V-O that precludes flame and oxygen reaching to the nanocomposites (Fig. 6e–g).

**Figure 7:**
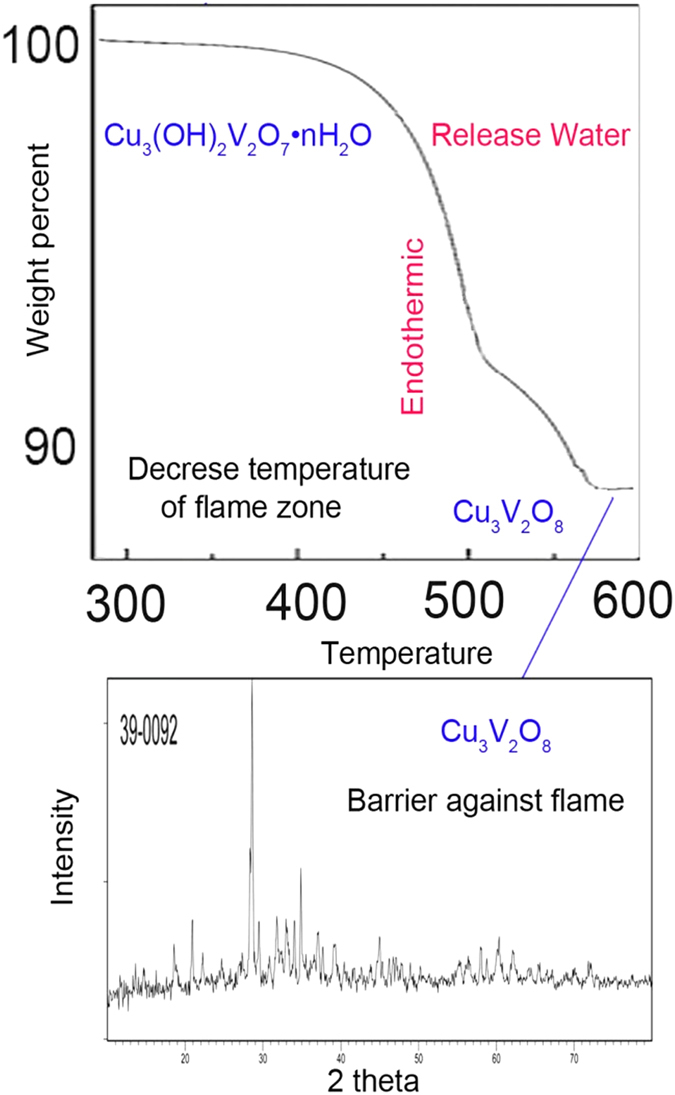
(**a**) TGA of Cu_3_V_2_O_7_(OH)_2_(H_2_O)_2_ and (**b**) conversion to Cu_3_V_2_O_8_ (**c**) DTA.

Dispersed nanoparticles play the role of a magnetic barrier layer^22^ which slows down product volatilization and prevents flame and oxygen from the sample during decomposition of the polymer. Cu_3_(OH)_2_V_2_O_7_·2H_2_O is converted to Cu_3_V_2_O_8_ with an endothermic reaction releases water and decreases temperature of the flame region (Fig. 7). Water vapour also dilutes flammable gases in the fire zone. In the presence of flame, magnetic nanoparticles remain together (show resistance to drop falling) and build a barrier.

Cu_3_V_2_O_7_(OH)_2_(H_2_O)_2_ is a hydrophilic product that show the best dispersion in water and as a results approve the best compatibility with hydrophilic PVA. Acetone as the second solvent for dispersing nanoparticles and dichloromethane has the worst compatibility with hydroxyls of the nanoparticles and as shown in SEM images agglomerated nanoparticles in the poly styrene matrix was observed.

Molar mass of Cu_3_V_2_O_7_(OH)_2_(H_2_O)_2_ is 474.5 g, at the first decomposition, 2H_2_O were evaporated from compound (36 g/474.5 g = 8%). At the second step of decomposition, another OH_2_ was evaporated (18 g/474 g.5 = 4%). The experimental results that were achieved from TGA analysis (8% weight loss at 450 °C and 4% weight loss at 550 °C = totally 12%) have suitable agreement with theoretical results for preparation of Cu_3_V_2_O_8_ (420.5 g/474.5 g = 88%) as a residual compound (Fig. 7b). Endothermic reaction was investigated and was confirmed by differential thermal analysis (DTA) and is shown in Fig. 7c. The flame retardancy could be a source of the char consisting of mainly Cu_3_V_2_O_8_ just blocking the air from reacting with the polymer. Actually flame retardancy in this work is result of synergism of barrier effect of nanostructure, release of water (3H_2_O: cooling the flame region) and endothermic decomposition (absorption the heating of flame zone)^36^. Also because of magnetic property of the nanostructures a compact and dense barrier is produced.

## Experimental

### Materials and Physical Measurements

CuSO_4_.5H_2_O, NH_4_VO_3_, ethylenediamine (en), propylenediamine (pn), triethylenetetramine (TETA) and tetraethylenepentamine (TEPA) were purchased from Merck Company. All of the chemicals were used as received without further purifications. For characterization of the products, X-ray diffraction (XRD) patterns were recorded by a Rigaku D-max C III, X-ray diffractometer using Ni-filtered Cu Ka radiation. Scanning electron microscopy (SEM) images were obtained on Philips XL-30ESEM. Transmission electron microscopy (TEM) image was obtained on a Philips EM208 transmission electron microscope with an accelerating voltage of 200 kV. Fourier transform infrared (FT-IR) spectra were recorded on Shimadzu Varian 4300 spectrophotometer in KBr pellets. The magnetic properties of the samples were detected at room temperature using a vibrating sample magnetometer (VSM, Meghnatis Kavir Kashan Co., Kashan, Iran). Room temperature photoluminescence (PL) was studied on a Perkin Elmer (LS 55) fluorescence spectrophotometer.

### Synthesis of Cu-organic complexes

An aqueous solution including 1 mol of CuSO_4_.5H_2_O in 50 mL of distilled water was added to a stoichiometric amount of ethylenediamine in 50 mL of distilled water under magnetic stirring. The mixture was stirred and heated (80 °C) for 5 h. The blue obtained precipitate was centrifuged, washed with ethanol and distilled water and dried at 50 °C. Other complexes were prepared via mentioned method.

### Synthesis of pure Cu_3_V2O_7_(OH)_2_(H2O)_2_ nanoparticles

First, 0.5 g of Cu-organic complex was dissolved into deionized water and was added to aqueous solution of NH_4_VO_3_ with a molar ratio of Cu:V = 3:2. After that, the above solution was heated at 100 °C and stirred for 1–2 h. The black precipitate was dried at 80 °C under vacuum for 2 h. In Scheme. 1, schematic diagram of formation of Cu_3_V_2_O_7_(OH)_2_(H_2_O)_2_ nanoparticles is depicted. The preparation conditions for synthesis Cu_3_V_2_O_7_(OH)_2_(H_2_O)_2_ nanoparticles have been illustrated in Table 1.

### Preparation of *ex-situ* nanocomposites

5 g of polymer was dissolved in 10 mL of solvent (25 °C) and then Cu_3_V_2_O_7_(OH)_2_(H_2_O)_2_ nanoparticles (1 g) was dispersed in 5 mL of solvent with ultrasonic waves (20 min, 60 W). Next, the dispersion of copper pyrovanadate was added slowly to the polymer solution. Solvent for poly styrene (PS), poly vinyl alcohol(PVA) and cellulose acetate (CA) are dichloromethane, water and acetone respectively. The solution was mixed under stirring for 6 h. For preparation of samples for UL-94 test after stirring, the product was casted on a template with dimension 130 × 13 mm and after about 48 h of solvent evaporation; the nanocomposite was placed in the vacuum oven for another 5 h for removal of residual traces of solvent. The final sheets for the test are 130 × 13 × 1.6 mm in dimension.

In UL-94 test a bar shape specimen of plastic 130 × 13 × 1.6 mm is positioned vertically and held from the top. A Bunsen burner flame is applied to the specimen twice (10 s each). A V-0 classification is given to material that is extinguished in less than 10 s after any flame application, drips of particles allowed as long as they are not inflamed. A V-1 classification is received by a sample with maximum combustion time <30 s, drips of particles allowed as long as they are not inflamed. The sample is classified V-2 if it satisfies the combustion time criteria of V-1, but flaming drips are allowed. Materials are ranked as N.C. in UL-94 tests when the maximum total flaming time is above 50 s. The sample is classified HB when slow burning on a horizontal specimen; burning rate <76 mm/min ^[28, 29].^

## Additional Information

**How to cite this article**: Ghiyasiyan-Arani, M. *et al.* Novel chemical synthesis and characterization of copper pyrovanadate nanoparticles and its influence on the flame retardancy of polymeric nanocomposites. *Sci. Rep.* **6**, 25231; doi: 10.1038/srep25231 (2016).

## Figures and Tables

**Table 1:** Reaction conditions for Cu_3_V_2_O_7_(OH)_2_(H_2_O)_2_ nanoparticles.

Sample No.	Cu precursore	Cu:V:amine molar ratio	Particle size (SEM) nm
1	[Cu(en)_2_]SO_4_	3:2:2	50–150
2	[Cu(pn)_2_]SO_4_	3:2:2	Agglomerated particles
3	[Cu(TETA)]SO_4_	3:2:1	Agglomerated particles
4	[Cu(TEPA)]SO_4_	3:2:1	10–150
